# Nogo-B is associated with cytoskeletal structures in human monocyte-derived macrophages

**DOI:** 10.1186/1756-0500-4-6

**Published:** 2011-01-14

**Authors:** Kathrin Schanda, Martin Hermann, Nadia Stefanova, Viktoria Gredler, Christine Bandtlow, Markus Reindl

**Affiliations:** 1 Clinical Department of Neurology, Innsbruck Medical University, Innsbruck, Austria; 2 KMT Laboratory, Department of Visceral-, Transplant- and Thoracic Surgery, Center of Operative Medicine, Innsbruck Medical University, Innsbruck, Austria; 3 Department of Neurobiochemistry, Innsbruck Medical University, Innsbruck, Austria

## Abstract

**Background:**

The reticulon Nogo-B participates in cellular and immunological processes in murine macrophages. Since leukocytes are an essential part of the immune system in health and disease, we decided to investigate the expression of Nogo-A, Nogo-B and Nogo-C in different human immune cell subpopulations. Furthermore, we analyzed the localization of Nogo-B in human monocyte-derived macrophages by indirect immunofluorescence stainings to gain further insight into its possible function.

**Findings:**

We describe an association of Nogo-B with cytoskeletal structures and the base of filopodia, but not with focal or podosomal adhesion sites of monocyte-derived macrophages. Nogo-B positive structures are partially co-localized with RhoA staining and Rac1 positive membrane ruffles. Furthermore, Nogo-B is associated with the tubulin network, but not accumulated in the Golgi region. Although Nogo-B is present in the endoplasmic reticulum, it can also be translocated to large cell protrusions or the trailing end of migratory cells, where it is homogenously distributed.

**Conclusions:**

Two different Nogo-B staining patterns can be distinguished in macrophages: firstly we observed ER-independent Nogo-B localization in cell protrusions and at the trailing end of migrating cells. Secondly, the localization of Nogo-B in actin/RhoA/Rac1 positive regions supports an influence on cytoskeletal organization. To our knowledge this is the first report on Nogo-B expression at the base of filopodia, thus providing further insight into the distribution of this protein.

## 1. Background

Reticulons (RTN) are ancient proteins mainly residing in the endoplasmic reticulum (ER) with diverse conserved physiological functions in membrane trafficking, apoptosis and cytoskeletal rearrangements/adhesion [1-6] as well as gain of function in certain tissues [7]. RTN4A/Nogo-A is the largest isoform encoded by the *rtn4/nogo gene*, which also gives rise to RTN4B/Nogo-B and RTN4C/Nogo-C [8,9]. Nogo-A is mainly expressed in white-matter oligodendrocytes where it acts as a neurite growth inhibitor [10] through interaction with the Nogo receptor (NgR) and the paired immunoglobulin-like receptor B (PirB) [11-13]. Nogo-C is found in neurons and skeletal muscle [14,15]. Recently, much attention is also drawn to the Nogo-B protein, the Nogo-B receptor (NgBR) and interaction partners [16-18]. Although Nogo-B is expressed ubiquitously, the highest expression is found in endothelial cells, vascular smooth muscle cells and inflammatory cells. In endothelial and smooth muscle cells, Nogo-B is associated with *in vitro *adhesion and promotion/inhibition of chemotaxis [19]. It has been demonstrated that Nogo-B knockout mice suffer from a lack of migrating tissue macrophages to sites of ischemic vascular injury and show slower wound healing [20]. Macrophages lacking Nogo-B present themselves with impaired Rac activation, spreading, migration, cytoskeletal organization and reduced expression of inflammatory genes upon stimulation. As leukocytes play an essential part in immunology, we investigated the expression and localization of Nogo-A, Nogo-B and Nogo-C in human immune cell subpopulations.

## 2. Results and discussion

### 2.1. Expression of Nogo splicing variants/isoforms in different populations of human immune cells

**Figure 1:**
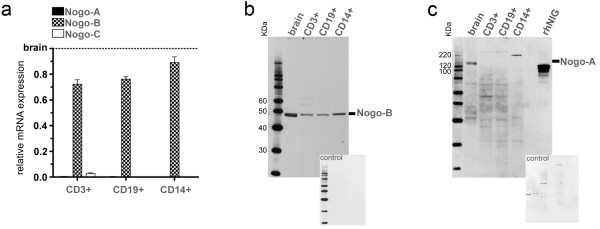
**Nogo expression in human immune cell subpopulations**. (a) Detection of high transcript levels of Nogo-B and very low levels of Nogo-A in human T cells (CD3+), B cells (CD19+) and monocytes (CD14+) in relation to the expression in human brain (human brain is set to the value of 1.0 on the y-axis). Only T cells express low levels of Nogo-C, whereas all Nogo splicing variants are highly expressed in human brain, serving as calibrator. Data are shown as means and standard deviations of independent real-time PCR experiments using cDNAs obtained from the respective immune cell populations of four different donors. (b) and (c) Representative Western Blots for the detection of Nogo-B and Nogo-A proteins in human brain/immune cell subpopulations and attached controls for unspecific reactions of secondary antibody. The protein distribution reflects the transcript level, with Nogo-B being present in all cell types and Nogo-A only showing signals in human brain at 140 KDa (the specific target peptide rhNIG serves as positive control). Nogo-B shows either two bands at 44/45 KDa in human brain, or one band at 45 KDa in immune cell subpopulations. Although the antibody used in (b) should be able to detect Nogo-C and Nogo-A, no signals were observed. Images shown are representative for independent experiments performed using the respective immune cell subpopulations of three different donors.

The expression of Nogo splicing variants/isoforms was analyzed in human monocytes (CD14+), T cells (CD3+) and B cells (CD19+). In a first step, mRNA expression was compared to the expression in human brain (Figure 1a). The levels of Nogo-A were very low (0.2-0.5% of brain expression), but present in all investigated cell populations. These findings are in line with studies demonstrating the role of Nogo-A in basic cellular functions, such as in shaping the membrane of tubular ER [21-23]. Nogo-C could only be detected in T cells (1.7-4.6% of brain expression), confirming its predominant expression in skeletal muscle and brain. In contrast, Nogo-B mRNA was strongly expressed in all cell populations, reaching 72% (T cells), 76% (B cells) and 89% (monocytes) of the expression in brain. This reflects its previously reported ubiquitous expression pattern [8,14,19,24].

To show whether mRNA levels were reflected on the protein level, western blots were performed. We detected Nogo-A and Nogo-B protein levels corresponding to their mRNA distribution pattern. Nogo-B presented itself with one 45 KDa band in all immune cell populations and two bands at 44 KDa and 45 KDa in human brain (Figure 1b), whereas Nogo-A was detectable at 140 KDa (Figure 1c). The molecular weights of human Nogo-A and Nogo-B correspond well to previous reports [25,26].

### 2.2. Nogo-B localization in human monocyte-derived macrophages depends on their morphology

**Figure 2:**
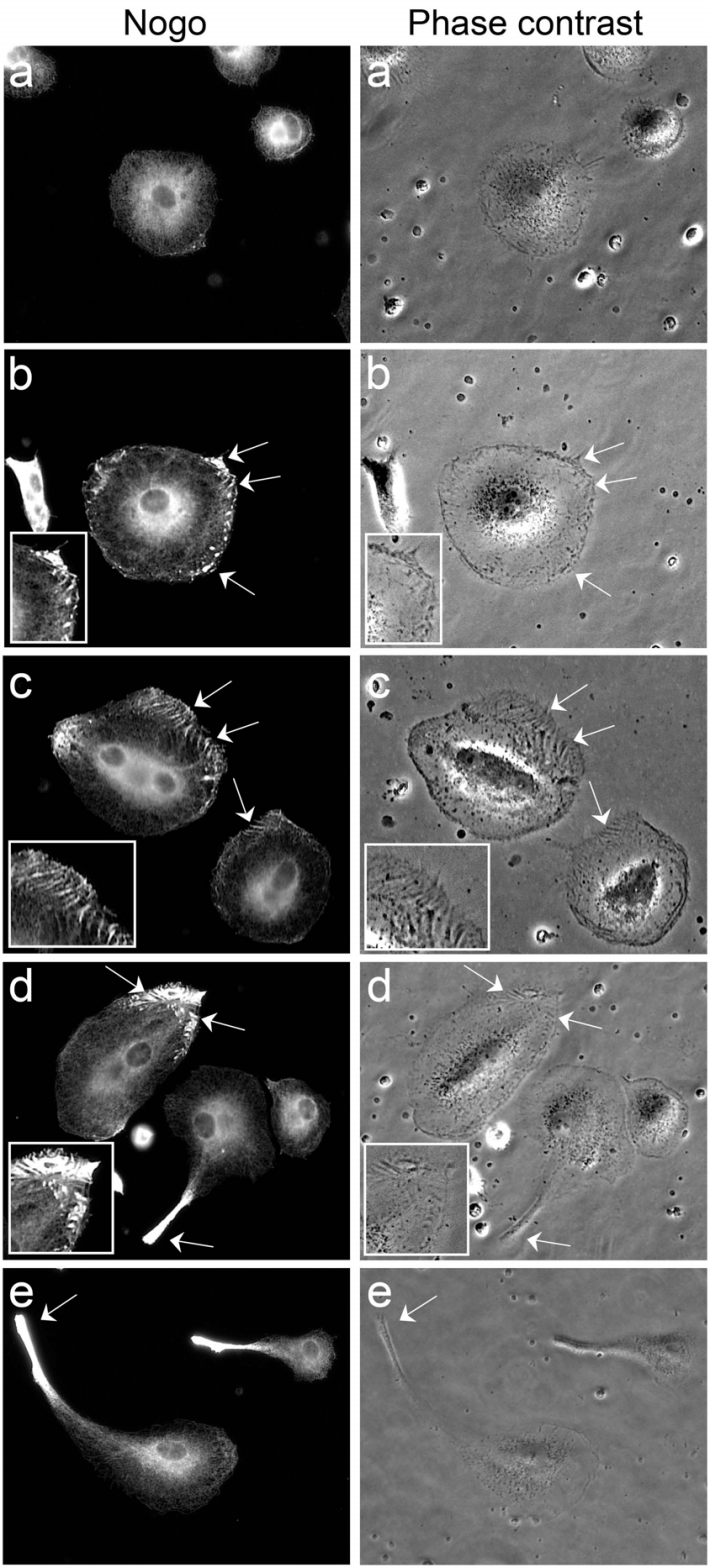
**Localization of Nogo-B in macrophages with different morphology**. Monocyte-derived macrophages showing different morphologies and the respective Nogo-B staining pattern (left panel). Structures similar to the staining pattern are visible in phase contrast microscopy (right panel): (a) Quiescent, round cell with reticular Nogo-B staining. (b) Activated cell with elongate and mainly punctate Nogo-B positive areas in the periphery (arrows, see inlay). (c) Radial, elongate Nogo-B staining patterns in the cell periphery of an elongated cell (arrows, see inlay). Note the unstructured Nogo-B accumulation in the left area of the cell. (d) Polarized, longish Nogo-B structures in an elongated cell (arrows, see inlay) (e) Strong Nogo-B translocation to the trailing end of migrating macrophages (arrow) with remnant reticular staining. All representative cell images are of one donor, showing comparable staining patterns observed in independent cultures of six donors. All images were acquired by conventional fluorescence microscopy using a DMI 4000B microscope and Application Suite V3.1 by Leica and represent cells cultured in 24-well cell culture plates.

In a next step, we analyzed the localization of Nogo-B in human monocyte-derived macrophages. Nogo-B showed different staining patterns depending on cell morphology (Figure 2).

In round and quiescent cells, Nogo-B showed the expected reticular staining pattern (Figure 2a) [Additional file 1]. However, towards elongated or activated cell shapes, an elongate and/or punctate Nogo-B pattern appeared in the cell periphery. Similar structures were observed in phase contrast microscopy (Figure 2b). Nogo-B positive, radial patterns in the cell periphery were possibly associated with the polarization level of the cell, showing a shift from widely spread distributions (Figure 2c) to a more polarized localization in cells with advanced elongation (Figure 2d). Finally, teardrop shaped and polarized, migrating macrophages showed a strong translocation of Nogo-B to the rear of the cell with remnant reticular staining (Figure 2e). The observations presented here possibly represent a dynamic change of Nogo-B localization during cytoskeletal changes or polarization [27-31].

### 2.3. Nogo-B is associated with cytoskeletal structures and Rho GTPases

**Figure 3:**
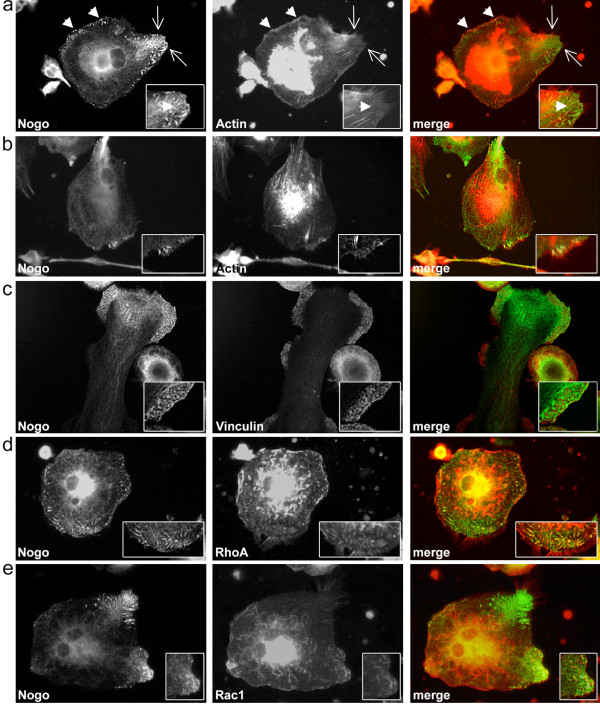
**Nogo-B and cytoskeletal structures in monocyte-derived macrophages**. (a) Partial co-localizations of Nogo-B and actin in punctate and elongate peripheral cell structures (arrowheads) and at the base of filopodia (arrows) with enlarged detail in inlay. Note that Nogo-B is not always present at the base of filopodia. (b) Nogo-B expression at the base of filopodia (see inlay). Actin stress fibers are not positive for Nogo-B. (c) Nogo-B is excluded from focal and podosomal adhesion sites (see inlay) (d) Intracellular, longish staining patterns of Nogo-B partially co-localize with RhoA (see inlay) (e) Nogo-B is present in Rac1 positive peripheral membrane ruffles (see inlay). Cells shown are representative of regularly observed staining patterns/co-localizations in independent macrophage cultures of six different donors. All images were acquired by conventional fluorescence microscopy using a DMI 4000B microscope and Application Suite V3.1 by Leica and represent cells cultured in 24-well cell culture plates.

Due to the observed staining patterns of Nogo-B shown above, a possible association with cytoskeletal elements might be postulated. Co-labeling with actin showed a partial co-localization in the area of elongate and punctate Nogo-B positive structures in the cell periphery (Figure 3a), whereas vinculin showed no co-labeling. In fact, vinculin positive focal/podosomal adhesion sites were exclusively excluded from the Nogo-B staining (Figure 3c). We propose that Nogo-B is possibly involved in cytoskeletal rearrangements, but not directly associated with adhesion sites. According to Yu et al [20], murine Nogo knockout macrophages show less actin expression and cell spreading than wildtype cells. In our study, punctate Nogo-B stainings were regularly observed at the base of long, thin, actin containing cell protrusions (Figure 3b). However, this observation did not apply to all such structures present on a cell (Figure 3a), possibly distinguishing between filopodia and retraction fibers, although the latter are often called filopodia in literature [32]. Filopodia are generally thought to act as sensors in exploring the extracellular matrix [33,34]. However, the view on filopodia function has expanded recently [35,36]. Xue et al [37] demonstrated an influence of filopodia development and presence on cell migration. Thus, the observed effect of less spreading and migration in Nogo knockout cells [20] may also be explained by its lack at the base of filopodia and the resulting effect on the cytoskeleton in general. Filopodia play an important role in chemotaxis, cell-cell interaction and pathway activation [38-41]. Therefore, the reduction of inflammatory cytokines upon stimulation in Nogo knockout macrophages observed by Yu et al [20] might also be explained by an impaired function of filopodia.

Yu et al explain the effect of reduced spreading and actin expression by showing low Rac activity in Nogo knockout cells as well as co-localizations of Nogo and Rac. As the actin remodeling factors Rac1 and RhoA are in close cross-talk [42,43] we decided to analyze their localizations in respect to Nogo-B. Therefore, co-labelings were performed, showing a partial co-localization with RhoA in elongate Nogo-B positive structures (Figure 3d). Rac1, as well as RhoA positive dorsal membrane ruffles were negative for Nogo-B (Figure 3d and 3e) although Rac1 positive peripheral membrane ruffles could be associated with Nogo-B (Figure 3e), as described before [20].

**Figure 4:**
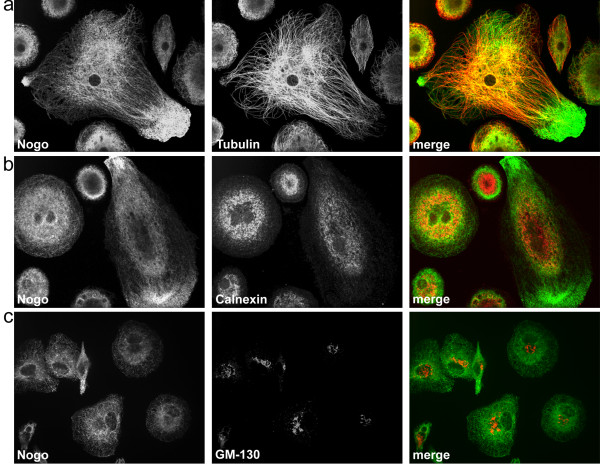
**Confocal imaging of Nogo-B and the tubulin network, endoplasmic reticulum and Golgi apparatus**. (a) Shows the strong association of Nogo-B with the tubulin network. Note the Nogo-B accumulation and detachment from tubulin in the cell protrusion. (b) Nogo-B co-localizes with the endoplasmic reticulum in the cell center, whereas no enriched calnexin signal can be observed in peripheral areas of Nogo-B accumulation. (c) Nogo-B shows a low presence in the Golgi apparatus compared to surrounding structures. All images are representative of regularly observed staining patterns/co-localizations in independent macrophage cultures of six different donors. Cells were analyzed by confocal laser scanning microscopy using an UltraVIEW RS (Perkin Elmer) mounted on an Olympus IX-70 inverse microscope. Images were acquired using the UltraVIEW RS software (Perkin Elmer). Shown are z-stacks of 0.5 μm thickness.

Tubulin co-labeling showed a strong co-localization with Nogo-B, supporting the observations by Rousseau et al [44]. Interestingly, the association with tubulin vanished in the area of Nogo-B accumulations in membrane protrusions (Figure 4a), indicating the detachment of Nogo-B from tubulin.

Nogo-B is a reticulon and localizes to the ER. Therefore, co-labelings with the ER residing protein calnexin showed an expected co-localization with Nogo-B in the cell center. However, no enriched calnexin signal was observed in the peripheral areas of strong Nogo-B accumulation (Figure 4b). This observation might be explained by the predominant localization of calnexin to rough ER [45], which is usually comprised of membrane sheets [46]. Therefore, an association of Nogo-B with tubular ER cannot be excluded in the Nogo-B enriched, peripheral areas.

Additionally, Golgi stainings with the marker GM-130 were performed. A possible association with Nogo-B could not be excluded, although Nogo-B does not seem to be accumulated in the Golgi area (Figure 4c).

## 3. Conclusion

The role of Nogo-B in monocyte-derived macrophages seems very diverse and challenging. In this study we demonstrate that Nogo-B is always present in the ER but can be translocated to large cell protrusions or the rear of migratory cells, where detachment from tubulin seems to take place. Additionally or independently, Nogo-B can be found at the base of filopodia, but not at focal/podosomal adhesion sites. Cells possibly undergoing polarization or cytoskeletal rearrangements show a prominent pattern of elongate or punctate Nogo-B positive areas in the periphery. Partial co-labelings with actin and the Rho GTPases RhoA and Rac1 support a direct or indirect influence on the cytoskeletal regulation. Furthermore, the finding of Nogo-B positivity at the base of filopodia may provide important information on these structures and gives deeper insight into a protein, which has not been associated in this context before.

## 4. Materials and methods

### 4.1. Isolation of peripheral blood mononuclear cells (PBMC)

Anonymized blood samples from 10 different healthy blood donors, which were not suitable for blood transfusion, were obtained with informed consent from the Central Institute for Blood Transfusion and Immunological Department (Innsbruck University Hospital) according to the ethical regulations of Innsbruck University Hospital. PBMC were isolated by density-gradient centrifugation, washed and resuspended in the respective medium or buffer.

### 4.2. Positive selection of immune cell subpopulations

Isolation was performed according to the manufacturers' instructions (BD Biosciences) using magnetic beads labeled with anti-CD3, anti-CD19 or anti-CD14 antibodies. After isolation, cells were counted and lysed with TRIZOL reagent (Invitrogen) or protein lysis buffer (1% NP-40, 150 mM NaCl, 50 mM HEPES pH 7.6, 0.8 mM MgCl_2_, 5 mM EGTA, protease inhibitor cocktail by Roche Applied Science). Purity assessed by flow cytometry was >89% in all cases (data not shown).

### 4.3. Semi-quantitative real-time PCR

RNA was extracted from the respective immune cell populations of four different donors with TRIZOL reagent according to the manufacturers' instructions. Samples and human brain RNA (Invitrogen AM6050) were transcribed to cDNA (SuperScript First-Strand Synthesis System for RT-PCR, Clontech Laboratories). Semi-quantitative real-time PCR was done with the SYBR^®^Advantage^®^qPCR Premix (Clontech Laboratories) using samples and standard concentrations of human brain cDNA in duplicates. The used primer pairs (Microsynth) and 7300 Applied Biosystems Cycler programming can be viewed in the additional files [Additional file 2]. Calculations were performed using the standard curve. Statistical analysis (means, standard deviations) was done using GraphPad Prism 5.

### 4.4. SDS-PAGE and Western Blot

Protein content of the respective immune cell lysates obtained from three different donors was determined using the BCA method (Sigma). The human brain tissue lysate was purchased from Abcam (ab29466). SDS-PAGE and Western Blot were performed with the NuPAGE system (Invitrogen) and the ECL Advance Kit (GE Healthcare), both used according to the manufacturers' instructions. After blocking, primary antibodies were added: goat anti-Nogo-A (Santa Cruz sc-11032) and rabbit anti-Nogo A/B/C (Chemicon AB5666P). Membranes were washed extensively, followed by incubation with the secondary antibody: anti-goat HRP (Santa Cruz sc-2354) or anti-rabbit HRP (GE Healthcare NA934). After washing, detection/exposure took place using films by GE Healthcare and the developer/fixer by Kodak. Successful quenching experiments with target peptides were performed (data not shown).

### 4.5. Cell culture and indirect immunofluorescence staining

PBMC of six different donors were individually resuspended in RPMI 1640 with Glutamax (Invitrogen) and cultured in cell culture plates (TPP) or on pre-coated Collagen I culture slides (BD Bioscience). Monocytes were allowed to adhere for 1 hour (37°C, 5% CO_2_), followed by the removal of non-adherent cells. Adherent cells were kept in culture for 8-10 days to generate mature monocyte-derived macrophages. The media used were CellGro DC medium (CellGenix) for cultures in cell culture plates (conventional fluorescence microscopy) or 5% autologous heat-inactivated serum in RPMI1640 with Glutamax containing antibiotics (Penicillin-Streptomycin, Invitrogen) for cultures on slides (confocal scanning microscopy). Cells for actin stainings were treated with 100 ng/ml Interferon-gamma (Invitrogen) for 48 h before fixation to enhance the actin staining. Cells were fixed/permeabilized with either 100% methanol (-20°C) or 4% paraformaldehyde (37°C)/0.2% CHAPS (Sigma). After blocking (5% normal goat serum, 1% bovine serum albumin, 2 μg/ml human immunoglobulin G in PBS, all by Sigma), the primary antibodies were added [see Additional file 3]. After washing, the secondary antibodies (Invitrogen) or TRITC-labeled phalloidin (Millipore) were added: anti-mouse Alexa Fluor 546, anti-rabbit Alexa Fluor 488.

Cells were analyzed by conventional fluorescence microscopy using a DMI 4000B inverse microscope and Application Suite V3.1 by Leica (Figure 2, Figure 3 and Additional file 1).

Cells were analyzed by confocal laser scanning microscopy using an UltraVIEW RS (Perkin Elmer) mounted on an Olympus IX-70 inverse microscope. Images were acquired using the UltraVIEW RS software (Perkin Elmer) in combination with a 100x oil immersion objective with a numerical aperture of 1.4. Excitation wavelenghts used were 488 nm (green) and 568 nm (red). Shown are z-stacks of 0.5 μm thickness. All images were edited using Adobe Photoshop CS3. Manipulations did not change the data content.

## Competing interests

The authors declare that they have no competing interests.

## Authors' contributions

KS carried out the indirect immunofluorescence stainings, performed the isolation of cells and Real-time PCR/Western Blot analysis and drafted the manuscript. MH took the pictures on the confocal microscope. NS participated in the design of the study and helped to draft the manuscript. VG carried out all cell culture methods. CB participated in the design of the study. MR conceived of the study, participated in the design of the study and carried out statistical analysis. All authors read and approved the final manuscript.

## Supplementary Material

Additional file 1 **Indirect immunofluorescence staining for Nogo-B in monocyte-derived macrophage cultures of different donors**. Images of individual mature monocyte-derived macrophage cultures obtained from a total of six different healthy human donors showing comparable Nogo-B staining patterns and distributions: cells show elongate-punctate, peripheral Nogo-B staining patterns as well as Nogo-B accumulations in membrane protrusions and at the trailing end of migrating macrophages. (a) Overview images, each from two separate donors. (b) Detail images, using anti-rabbit Alexa Fluor 546 antibodies (Invitrogen) for detection; images are acquired from stainings of independent cultures obtained from three different donors. (c) Detail images, using anti-rabbit Alexa Fluor 488 antibodies (Invitrogen) for detection; images are acquired from stainings of independent cultures obtained from five different donors; scale bar is 20 μm; all images were acquired by conventional fluorescence microscopy using a DMI 4000B microscope by Leica with either Application Suite V3.1 by Leica or a mounted Canon Power Shot A620 digital camera. Click here for file

Additional file 2 **Primers used for detection of Nogo splicing forms/housekeeping genes**. Table of primer pairs used for real-time PCR and GenBank transcript accession number of human Nogo-A (RTN4A), Nogo-B (RTN4B), Nogo-C (RTN4C) as well as ACTB (beta actin) and TBP (TATA box binding protein), serving as housekeeping genes for analysis. The thermo cycler was programmed as follows: 2 min at 95°C, followed by 40 cycles (10 sec at 95°C and 31 sec at 68°C). As quality control, a melting curve was added after each run. File can be viewed in landscape format. Click here for file

Additional file 3 **Primary antibodies used in indirect immunofluorescence co-labeling**. Antibodies used in co-labeling of Nogo-B and Vinculin (focal/podosomal adhesion sites), RhoA and Rac1 (cytoskeletal structures), Tubulin (tubulin network), Calnexin (endoplasmic reticulum) and GM-130 (Golgi apparatus) in monocyte-derived macrophages. Click here for file
