# Supplementary figures and images for: Nogo-B is associated with cytoskeletal structures in human monocyte-derived macrophages
Source: BMC Res Notes. 2011 Jan 14;4:6. doi: 10.1186/1756-0500-4-6 (PMC3029212; doi:10.1186/1756-0500-4-6)

a

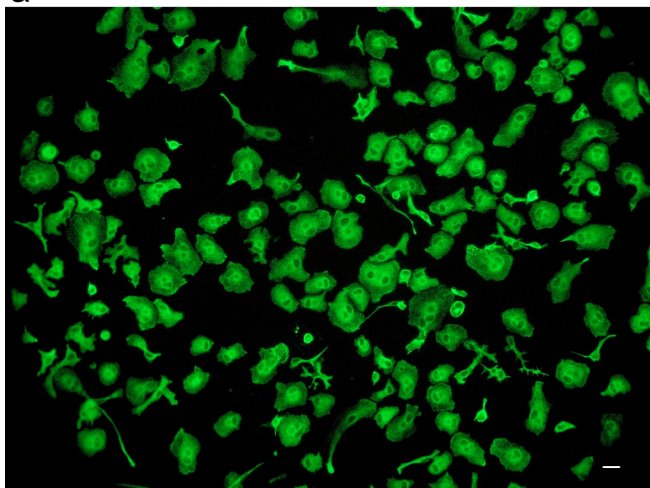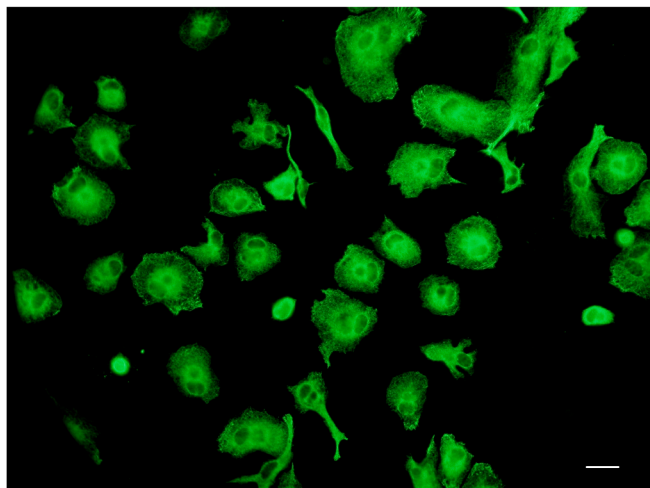

b

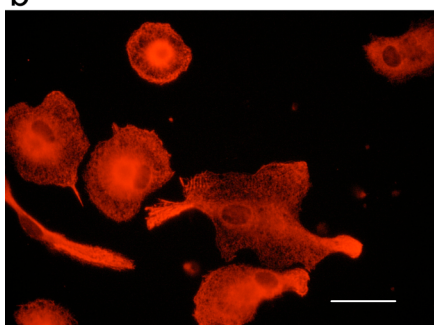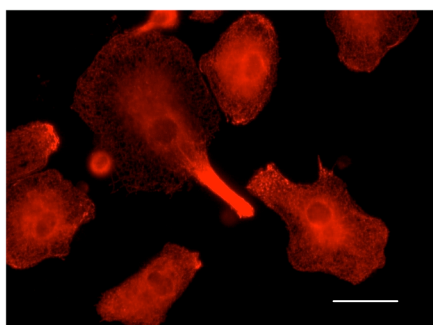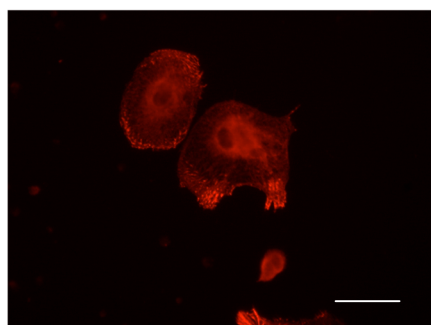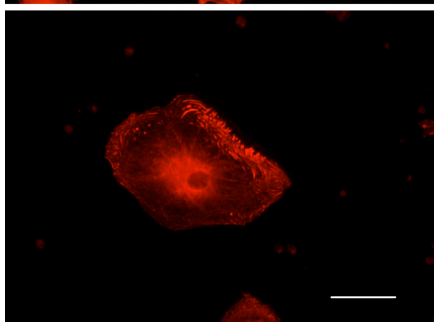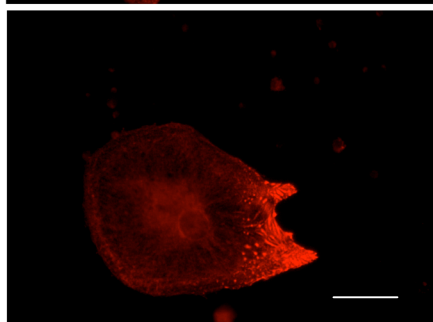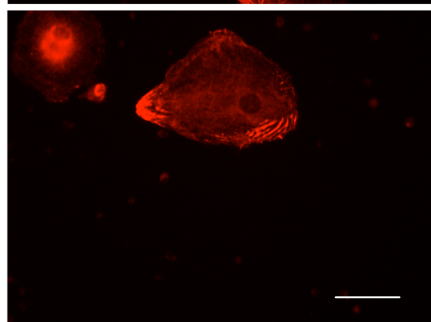

c

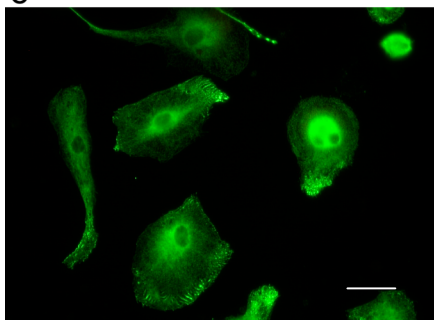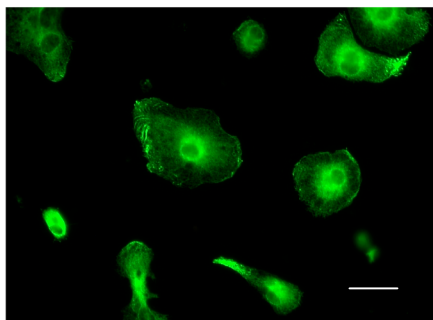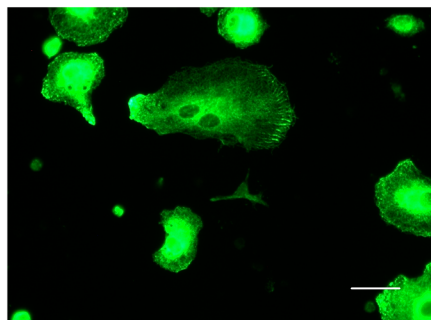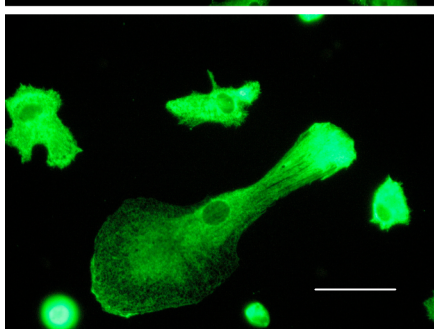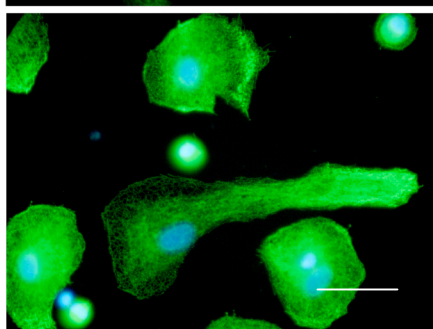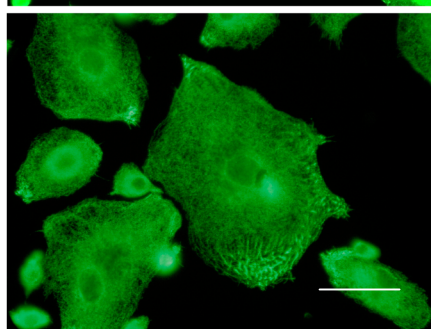

Supplement: Additional file 1 — Indirect immunofluorescence staining for Nogo-B in monocyte-derived macrophage cultures of different donors. Images of individual mature monocyte-derived macrophage cultures obtained from a total of six different healthy human donors showing comparable Nogo-B staining patterns and distributions: cells show elongate-punctate, peripheral Nogo-B staining patterns as well as Nogo-B accumulations in membrane protrusions and at the trailing end of migrating macrophages. (a) Overview images, each from two separate donors. (b) Detail images, using anti-rabbit Alexa Fluor 546 antibodies (Invitrogen) for detection; images are acquired from stainings of independent cultures obtained from three different donors. (c) Detail images, using anti-rabbit Alexa Fluor 488 antibodies (Invitrogen) for detection; images are acquired from stainings of independent cultures obtained from five different donors; scale bar is 20 μm; all images were acquired by conventional fluorescence microscopy using a DMI 4000B microscope by Leica with either Application Suite V3.1 by Leica or a mounted Canon Power Shot A620 digital camera. [file 1756-0500-4-6-S1.PDF]
