# Supplementary material for: Nogo-B is associated with cytoskeletal structures in human monocyte-derived macrophages
Source: BMC Res Notes. 2011 Jan 14;4:6. doi: 10.1186/1756-0500-4-6 (PMC3029212; doi:10.1186/1756-0500-4-6)
Supplement: Additional file 2 — Primers used for detection of Nogo splicing forms/housekeeping genes. Table of primer pairs used for real-time PCR and GenBank transcript accession number of human Nogo-A (RTN4A), Nogo-B (RTN4B), Nogo-C (RTN4C) as well as ACTB (beta actin) and TBP (TATA box binding protein), serving as housekeeping genes for analysis. The thermo cycler was programmed as follows: 2 min at 95°C, followed by 40 cycles (10 sec at 95°C and 31 sec at 68°C). As quality control, a melting curve was added after each run. File can be viewed in landscape format. [file 1756-0500-4-6-S2.PDF]

**Transcript**	**GenBank**	**Forward primer**	**Reverse primer**
Nogo-A	NM_020532	TCCTCGGGCTCAGTGGATGAGA	GGAGAGACACTGAACGATGATCCCA
Nogo-B	NM_153828	TCCTCGGGCTCAGTGGTTGTTG	TGCCCTGAATGGGTGGCCTT
Nogo-C	NM_007008	CGGTCAGAAGAAAAATTGGAAGGAC	CGTGCAGTTCACATGACCAAGAGCA
ACTB	NM_001101	TGTGCTACGTCGCCCTGGACTTCGA	AATGCCAGGGTACATGGTGGTGCCG
TBP	NM_003194	GGTGCCATGACTCCCGGAATCCCTA	CCTGAGGTTCCCTGTGTTGCCTGCT
