# Supplementary material for: Nogo-B is associated with cytoskeletal structures in human monocyte-derived macrophages
Source: BMC Res Notes. 2011 Jan 14;4:6. doi: 10.1186/1756-0500-4-6 (PMC3029212; doi:10.1186/1756-0500-4-6)
Supplement: Additional file 3 — Primary antibodies used in indirect immunofluorescence co-labeling. Antibodies used in co-labeling of Nogo-B and Vinculin (focal/podosomal adhesion sites), RhoA and Rac1 (cytoskeletal structures), Tubulin (tubulin network), Calnexin (endoplasmic reticulum) and GM-130 (Golgi apparatus) in monocyte-derived macrophages. [file 1756-0500-4-6-S3.PDF]

Target	Host	Dilution	Company	Part number
Nogo-A/B	rabbit	1:1000	Abcam	ab47085
Vinculin	mouse	1:100	Millipore	90227 (7F9)
RhoA	mouse	1:400	Abcam	ab54835
Rac1	mouse	1:400	BD Transduction	610651
Tubulin	mouse	1:500	Sigma	T5168
Calnexin	mouse	1:100	Abcam	ab31290
GM-130	mouse	1:500	BD Transduction	610822
